# The Influence of Culture on Attitudes Towards Humorous Advertising

**DOI:** 10.3389/fpsyg.2019.01015

**Published:** 2019-05-08

**Authors:** Yi Wang, Su Lu, Jia Liu, Jiahui Tan, Juyuan Zhang

**Affiliations:** ^1^Business School, Central University of Finance and Economics, Beijing, China; ^2^Business School, University of International Business and Economics, Beijing, China

**Keywords:** culture, humor, brand, humorous advertising, advertising tactics

## Abstract

Humor has been widely used in advertising in recent decades. Various studies found that humor could significantly improve advertising performance. However, most of these studies were conducted in a Eastern context and did not consider cultural factors. In a cross-cultural research framework, the current study explored the effects of advertisement characteristics (i.e., brand nationality and humor tactics) on Chinese and United States audiences’ attitudes toward humorous advertisements. Results showed that the attitudinal differences between Chinese audiences and United States audiences was not significant at the aggregate level. Instead, the differences lie in an audience’s responsiveness to characteristics of the ads. Specifically, while United States audiences showed a strong preference for ads featuring Chinese brands compared to those of United States brands, Chinese audiences did not differentiate them. United States audiences preferred ads using self-enhancing tactics to those using affiliative tactics, whereas, again Chinese audiences did not differentiate. We also explored whether individual differences in cultural values could account for the effect of audience nationality. Results suggest that differences embedded in culture groups, as indicated by audience nationality, could not be explained or substituted by individual variance in humor tolerance and uncertainty avoidance. Limitations and future directions were discussed.

## Introduction

Every year, Clio Awards, the Oscars of advertising, present an award named, “Best Use of Humor” (Buijzen and Valkenburg, 2004). This shows that not only have using humor been a common practice in the advertising business, but also been highly recognized of its economic values in commercials. Indeed, in the recent years, using humor in advertising has become a common practice worldwide (Duncan, 1979). Nearly 36% of TV commercials in United Kingdom use humor appeals, and 24% of TV commercials, 31% of radio advertisements and 15% of magazine advertisements in United States contain elements of humor (Eisend, 2018).

In China, the application of humorous advertising seems to be conservative and limited. For example, there is a popular lemon drink advertisement conducted by a very famous American brand. In this printed advertisement, in order to show the original taste and flavor of the drink, an anthropomorphic lemon is standing on the edge of the cup and peeing into the lemon drink. It has achieved good communication effects among Western audiences, but many Chinese audiences feel uncomfortable about it.

This discomfort exists because people in different cultures hold strikingly different attitudes toward humor (Kuiper et al., 2010; Yue et al., 2016) and humorous advertising (Hatzithomas et al., 2011). Whereas Western audiences hold a positive attitude toward humor, Chinese audiences hold a subtle negative attitude toward humor (Yue, 2010). Consequently, in Western countries, people regard humor as a common and positive disposition for everyone, and humorous advertisement has been used in marketing practices in all kinds of media (Weinberger and Gulas, 1992). While in China, people view humor as a personality trait possessed exclusively by specialists in humor-related fields (Yue, 2010), and only a quarter of Chinese commercial advertisements use humorous elements (Hu and Huang, 1998), most of which appear in emerging online advertisements (Zhou, 2008).Despite the prevalence of humor appeals in the advertising industry, little attention has been paid to the attitudinal differences of humorous advertisements in a cross-cultural framework (Hatzithomas et al., 2011), meaning we know little of how characteristics of an ad interact with cultural factors. Do audiences from different cultural contexts hold different attitudes toward the same humorous advertisement? If yes, could these differences be explained by individual differences concerning cultural values? To answer the above questions, we compared evaluations of audiences from different cultures concerning advertisements varying in brand nationalities and tactics.

## Literature Review

Although the cultures of the West and East are similar in humanistic structure and value, they hold different attitudes toward humor (Alden et al., 1993). People from a western maritime culture are open and forthright, and they regard humor as a common positive and optimistic character, which is usually associated with positive words (Yue et al., 2016). On the other hand, people from Chinese culture are deeply influenced by traditional Confucianism, are reserved, advocate the golden mean, and consider public humor as less elegant and decent (Jiang et al., 2011; Yue, 2010). This leads to their differences in attitudes toward humorous advertising.

### Attitudes Toward Humorous Advertising

Since the 1960s, American companies have come to realize that audiences deeply resent the intrusion of advertising and marketing interruptions and that these could be ameliorated by humor factors, which eliminate prejudice and alarm, induce positive moods, and convey advertising purposes unwittingly (Spotts et al., 1997). As a result, humorous advertising received more and more attention from academia. Sternthal and Craig (1973) first explored the use of humor in advertising and found that humor increased audiences’ attention and advertising performance. Scott et al. (1990) argued that humor could induce a pleasant cognitive or emotional response by comparing the expected with the unexpected. Beard (2005) suggests that a proper humorous advertisement can not only delight audiences, but also reduce audiences’ bad impression on the brand or product, attaining the goal of advertisements.

Humorous advertising can stimulate audiences’ positive sentiment and induce them into highly advertisement engagement, resulting in a more positive attitude (Chattopadhyay and Basu, 1990; Eisend, 2011). Nonetheless, people from the Chinese culture do not think of humorous advertising as a creative marketing method (Yue, 2010). In a cross-cultural research framework of humorous advertising, the researchers noticed two interesting questions: Does the audience have different attitudes to humorous advertisements from different countries? Does the audience have different attitudes to humorous advertisements with different humorous advertising tactics? These two questions are widely considered by both advertising researchers and advertisers in the advertising tactics decision process.

#### The Role of Brand Nationality

Because of differences in culture, habits of thought, and market environments in China and the West, humorous advertisements have different styles and appeals (Hu and Huang, 1998). Research has noticed that in the dissemination of humorous advertisements, audiences have different advertising perceptions depending on brand nationality (Madden and Weinberger, 1982). Western humorous advertisements are relatively bold, and can subvert almost anything as tools of humor, whether the national flag or the President (Jiang et al., 2011). For example, Pepsi launched one such advertisement in which the queen of England jumped off a building and had a party with people, even after being thrown to the ground she continued the party as if nothing had happened.

Chinese humorous advertisements are relatively conservative, as in China such a subversive advertisement as Pepsi’s is legally forbidden. It is difficult for traditional Chinese people to accept the exaggeration and indecency in advertising because they do not meet the aesthetics and requirements of Chinese audiences and may even cause great dislike and resentment (Yue et al., 2016). The differences in styles and expressions between Chinese and Western humorous advertisements have gradually formed stereotypes about brand images, with the Chinese corporate image generally being more serious and lacking a sense of humor, while Western companies appear as being relatively easy and communicative in a humorous way (Chattopadhyay and Basu, 1990). Hence, we predict that:

H1: Audience’s attitude toward ads of Chinese brands is more positive than those of United States brands.
H2: Americans hold more positive attitude toward Chinese brands than United States brands. Chinese attitudes toward Chinese and United States brands differ less.

#### The Role of Humor Tactics

Another important factor that influences the audience’s attitudes toward humorous advertising is humorous advertising tactics. In different advertising scenarios, advertisers use different humorous advertising tactics to achieve the desired brand communication goals. Humorous tactics can be categorized into four types: affiliative, self-enhancing, self-defeating and aggressive. Given that the former two are more adaptive and trigger more positive emotions than the latter two (Martin et al., 2003), they are commonly used tactics in advertising practice.

Affiliative humorous ads focus more on others, and are generally used to entertain the audience. By using witty and “harmless” jokes, this type of humorous advertising can reduce the tension in advertising and make people smile when they see an advertisement. Affiliative humorous advertisements focus more on the harmonious relationship between brands and audiences, and entertain audiences by using witty and “harmless” jokes. They take advantage of the generally accepted method of ridicule, which can reduce the audiences’ nervousness and achieve the communication goals (Eisend, 2018).

Self-enhancing humorous ads tend to pay more attention to the advertising products. This type of ad can be understood as a “self-proclaiming” humorous advertisement. Through the enlargement and exaggeration of the brand benefit, the advertisements strengthen the brand position and promote the brand image (Scott et al., 1990). Due to the high acceptance of humor by Western consumers and the ubiquity of humor in daily life, the exaggerated expression and humor of self-improvement humorous expressions can make it easier to express their own brand or product performance.

Given that the four styles identified in Martin’s humor model were originally formulated in a North American individualistic context, we suggest that Western audiences could favor the self-enhancing humorous advertisements because of the exaggerated expressions focusing on the independent “self.” Eastern audiences from collective cultures, however, may differentiate these tactics to a lesser extent, given their interdependent self-construal blurring the boundaries between the self and others (Taher et al., 2008). Indeed, several cross-cultural comparisons show that American participants showed a distinctive positive reaction to self-enhancing humorous style, whereas their counterparts from a collectivistic culture did not show differentiation in their responses to self-enhancing and to affiliative humorous styles (Kazarian and Martin, 2004, 2006; Kuiper et al., 2010). Hence, we predict that:

H3: Americans hold more positive attitude toward ads using self-enhancing tactics than they do for ads using affiliative tactics. Chinese attitudes toward ads using affiliative tactics and self-enhancing tactics differ less.

#### The Role of Cultural Values

In identifying factors that might account for differences in perception and application of humorous advertisements in different countries, many scholars point to the role of cultural values (e.g., Alden et al., 1993).

One such factor is *humor tolerance*. As a sub-dimension of the sense of humor, humor tolerance refers to the extent to which one can tolerate taboos and off-limits topics as the object of humor (Herzog and Strevey, 2008; Yue et al., 2016). People high in humor tolerance regard humor as a natural expression of joy and an indispensable spice in a recreational, amusement, and social life. They use humor regardless of time, occasion and object of ridicule (Yue, 2010). Whereas low humor tolerance cultures emphasize rules and order in social relationships. Humor used in public occasions is deemed inappropriate and being ridiculed or joking in public is sometimes considered an offense (Jiang et al., 2011; Yue et al., 2016). Moreover, people from these cultures often think that humor is a characteristic of certain groups, such as comedians. Presenting humorous and interesting images in public does not match their status. What is more, humor is sometimes associated with negative vocabulary, such as shallowness and frivolity (Yue, 2010). Higher humor tolerance will lead one to pay more attention to the plot of the advertisement *per se*; whereas lower humor tolerance will lead one to pay more attention to the humorous stimuli in advertisements. Therefore, we predict that:

H4: Audiences higher in humor tolerance hold a more positive attitude toward ads using self-enhancing tactics than for ads using affiliative tactics. Attitudes of audience lower in humor tolerance toward ads using affiliative tactics and self-enhancing tactics differ less.

Another cultural factor that is particularly relevant to humor perception is *uncertainty avoidance*. Eisend (2011). Among Hofstede’s five national cultural dimensions, uncertainty avoidance is the extent to which people feel uncomfortable or threatened by uncertainty and the unknown (Hofstede, 2011). Studies have shown that uncertainty avoidance can influence the effectiveness of humorous advertising (De Mooij, 1998; Lee and Lim, 2008). Consumers with low uncertainty avoidance prefer humor-oriented advertising; whereas consumers with high uncertainty avoidance pay more attention to the advertising information (Hatzithomas et al., 2011). Compared with affiliative advertisements, self-enhancing advertisements are more informative concerning products’ core functions. Given that audiences high in uncertainty avoidance are motivated to get more reliable information about the product and brand to eliminate uncertainty (Hatzithomas et al., 2011), they should prefer self-enhancing advertisements. While audiences low in uncertainty avoidance pay more attention to the content of advertisements, rendering the effect of tactics less prominent. Therefore, we predict that:

H5: Audience higher in uncertainty avoidance prefer self-enhancing to affiliative advertisements. Audience lower in uncertainty avoidance differentiate less.

### Overview

Humor enhances ad related attitudes primary through affective routes (Eisend, 2009, 2011), such that humor triggers an immediate affective response, which then transfers to the ad and the brand (Gelb and Pickett, 1983; De Houwer et al., 2001; Strick et al., 2009). Indeed, a recent meta-analysis shows that humor enhances positive emotions, attitudes toward the ad, and attitudes toward the brand (Eisend, 2009). Therefore, in this research, we focused on the three key metrics of advertising attitudes, including positive emotion (an affective component), humor level of the ad (e.g., how humorous each ad is—a cognitive component), and consumer connections to brands (e.g., a strong indicator of behavioral intention).

Will audiences from different cultures evaluate the same humorous advertisement differently? If so, could these differences be accounted for by differences in cultural values? To these ends, we recruited participants from the United States and China that are representative of the Western and the Eastern cultural contexts, and conducted a cross-cultural comparison using a 2 (humorous tactics: affiliative, self-enhancing) × 2 (brand nationality: Chinese brand, United States brand) × (audience nationality: Chinese, United States) between-subject design. Furthermore, we considered whether individual differences concerning humor tolerance and uncertainty avoidance could account for cultural differences at the group level.

## Research Design

### Participants

A total of 506 participants were recruited in this experiment (234 Chinese and 272 United States). The Chinese participants were recruited from the biggest online Chinese survey website, wjx.com; the United States participants were recruited from American crowdsourcing marketplace, Amazon MTurk. Our study was approved by the Ethics Review Board of Business School of Central University of Finance and Economics. All participants signed an informed consent form.

Participants were randomly assigned to four experimental conditions. We controlled the IP address to ensure that each volunteer participant only answered the questionnaire once. The average answer time was 512 s after removing the cases with extremely short response time (less than 180 s). We removed invalid cases with the help of inverse coding questions and obtained 393 valid cases, including 230 Chinese and 163 United States participants.

### Materials and Pre-test

The experimental context was print advertisement. We collected 132 pre-test print advertisements from publications and websites and asked 39 graduate students to evaluate them in terms of levels of innovation, humor, and familiarity. Five graduate students separated them into affiliative advertisements and self-enhancing advertisements. Based on their responses, 6 advertisements were selected for their high consistency and distinctiveness. We then created 6 Chinese virtual brands and 6 United States virtual brands for the advertisements. To provide a realistic setting, the brand name for the advertisements were adapted from real brands, but identifiable characteristics of the brands were removed. At last, we replaced the original brands on the advertisement with the virtual brands using Photoshop and got 12 experimental advertisements, which belong to one of the 4 conditions in a 2 (humorous advertising tactics: affiliative, self-enhancing) × 2 (brand nationality: Chinese brand, United States brand) design, with 3 advertisements in each cell.

### Measures and Procedure

After participants watched the three experimental advertisements, they needed to respond to a survey that comprises of measurements of humor level (Herzog and Strevey, 2008), positive emotion (Philip etc., 2008), self-brand connection (Escalas, 2004), humor tolerance (Herzog and Strevey, 2008), and uncertainty avoidance (Hofstede and Bond, 1984). At last, they reported demographics information (e.g., age, gender, and income). All questions were adapted from existing mature scales with the form of a seven-point Likert scales (1 = “not at all,” and 7 = “extremely”). Whereas Chinese participants completed a Chinese version of the questionnaire, their United States counterparts completed an English version. Accuracy and equivalence of the translation was secured using multiple rounds of back-translation. Reliability analyses showed that all Cronbach’s α were greater than 0.7. We averaged the scores for positive emotion (an affective component), humorous level of the ad (a cognitive component), and consumer connections to brands (a indicator of behavioral intention) to indicate the attitudes toward humorous advertisement (Cronbach’s α = 0.869).

## Results

### Audience Nationality as Different Cultural Groups

We conducted a univariate analysis of covariance to test the prediction that the Chinese audience and the United States audience hold different attitudes toward humorous advertisements depending on humor tactics and brand nationality, controlling for age, gender, and income. Table 1 displays the results in detail.

**Table 1:** Description of Samples.

Chinese participants(*n* = 230)			United States participants(*n* = 163)		
Variable gender	*n*	%	Variable gender	*n*	%
Female ( = 0)^∗^	138	60.0	Female ( = 0)^∗^	83	50.9
Male ( = 1)	92	40.0	Male ( = 1)	80	49.1
**Age**			**Age**		
under 18 ( = 1)^∗^	1	0.4	under 18 ( = 1)^∗^	–	–
18–25 ( = 2)	62	27.0	18–25 ( = 2)	16	9.8
26–30 ( = 3)	68	29.6	26–30 ( = 3)	31	19.0
31–40 ( = 4)	75	32.6	31–40 ( = 4)	56	34.4
41–50 ( = 5)	16	7.0	41–50 ( = 5)	31	19.0
Above 51 ( = 6)	8	3.5	Above 51 ( = 6)	29	17.8
**Monthly income (¥)**			**Monthly income ($)**		
Under 5000 ( = 1)^∗^	72	31.3	Under 2000 ( = 1)^∗^	40	24.5
5001–10000 ( = 2)	108	47.0	2001–5000 ( = 2)	85	52.1
10001–20000 ( = 3)	43	18.7	5001–10000 ( = 3)	29	17.8
Above 20001 ( = 4)	7	3.0	Above 20001 ( = 4)	9	5.5

*(1)^∗^ Control group of category variables. (2) The frequency and percentage of variables are reported.*

Results showed that the main effect of brand nationality was significant, *F* (1,382) = 10.124, *p* < 0.01. Audiences held more positive attitudes toward Chinese brands than United States brands, confirming Hypothesis 1.

**FIGURE 1:**
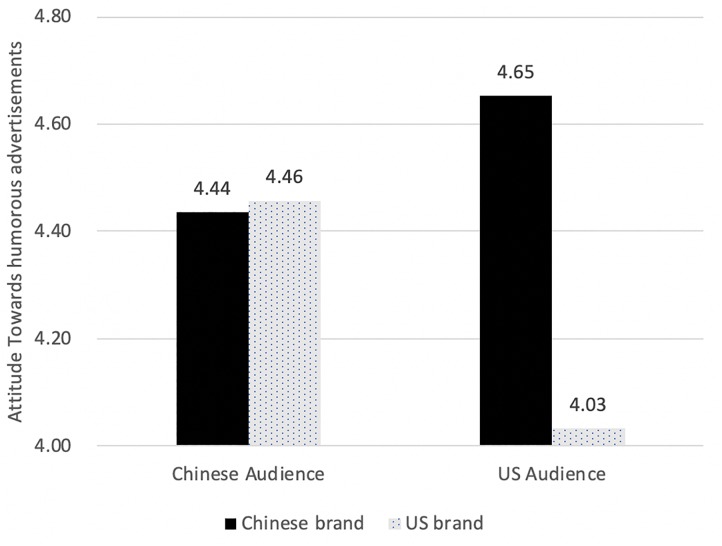
Attitude difference in brand nationality with different audience nationality.

As Hypothesis 2 suggested, the audience’s nationality significantly interacted with brand nationality *F* (1, 382) = 11.443, *p* < 0.01 (Figure 1). Simple main effect analyses showed that United States audience held more positive attitude toward Chinese brands than toward United States brands [*F*(1,382) = 18.30, *p* < 0.001]; whereas Chinese audiences did not differentiate, *F* (1,382) = 0.032, *p* > 0.05.

**FIGURE 2:**
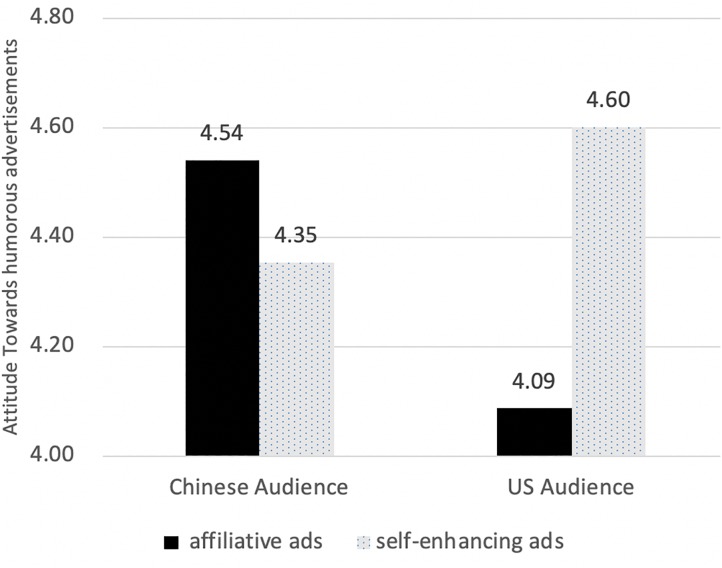
Attitude difference in advertising tactics with different audience nationality.

As Hypothesis 3 suggested, audience nationality significantly interacted with humor tactics, *F*(1,382) = 13.902, *p* < 0.001; (Figure 2). Simple main effect analyses showed that United States audience held more positive attitude toward self-enhancing ads than toward affiliative ads, *F*(1,382) = 12.629, *p* < 0.001; whereas Chinese audience did not differentiate. No other effects were significant.

### Humor Tolerance as a Cultural Moderator

Humor tolerance is an important cultural factor that influences the way audiences perceive and react to humorous advertisements. We categorized participants into high and low humor tolerant groups based on a mean level of humor tolerance. We conducted a univariate analysis of covariance to test the prediction that audiences with different humor tolerances hold different attitudes toward humorous advertisements depending on humor tactics and brand nationality, controlling for age, gender, and income. Table 2 displays the results in detail.

**Table 2:** Attitude model of humorous advertisement.

Dependent variable: Attitude toward humorous advertisements					

Source	Type III sum of squares	*df*	Mean square	*F*	Significant
Corrected Model	33.543^a^	10	3.354	4.014	0.000
Intercept	425.613	1	425.613	509.320	0.000
gender	0.243	1	0.243	0.290	0.590
age	0.768	1	0.768	0.919	0.338
incoming	6.276	1	6.276	7.510	0.006
**Brand nationality**	**8.460**	**1**	**8.460**	**10.124**	**0.002**
Advertising tactics	2.502	1	2.502	2.994	0.084
Audience nationality	0.889	1	0.889	1.064	0.303
Brand nationality ^∗^ Advertising tactics	0.285	1	0.285	0.341	0.560
**Brand nationality ^∗^ Audience nationality**	**9.562**	**1**	**9.562**	**11.443**	**0.001**
**Advertising tactics ^∗^ Audience nationality**	**11.617**	**1**	**11.617**	**13.902**	**0.000**
Brand nationality ^∗^ Audience nationality ^∗^ Audience nationality	0.951	1	0.951	1.139	0.287
Error	319.218	382	0.836		
Total	7965.847	393			
Corrected Total	352.761	392			

*a. R Squared = 0.095 (Adjusted R Squared = 0.071).*

Results showed that the main effect of brand nationality was significant, *F* (1,382) = 7.722, *p* < 0.01. Audiences held a more positive attitude toward Chinese brands than United States brands, confirming Hypothesis 1.

**FIGURE 3:**
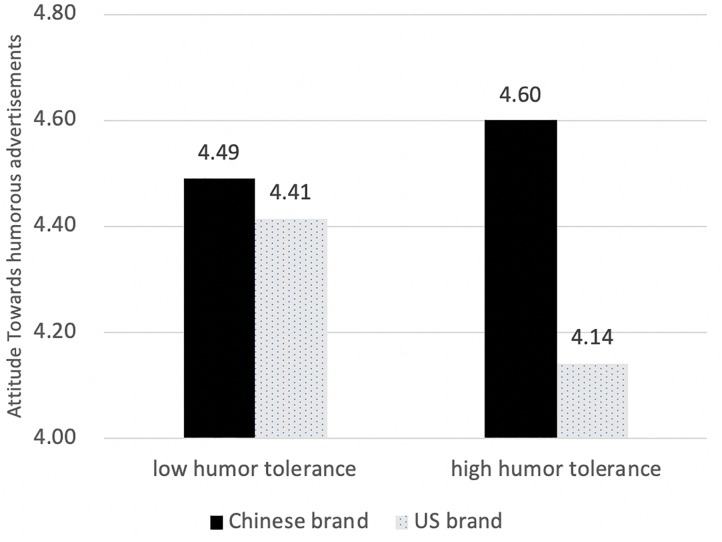
Attitude difference in brand nationality with different levels of humor tolerance.

As Hypothesis 4 suggested, humor tolerance significantly interacted with humor tactics *F*(1,382) = 4.805, *p* < 0.5 (Figure 3). Simple main effect analyses showed that audiences high in humor tolerance held a more positive attitude toward humorous ads than audiences low in humor tolerance [*F*(1,382) = 0.534, *p* < 0.001]; whereas an audience low in humor tolerance did not differentiate, *F*(1,382) = 0.384, *p* > 0.05.

**FIGURE 4:**
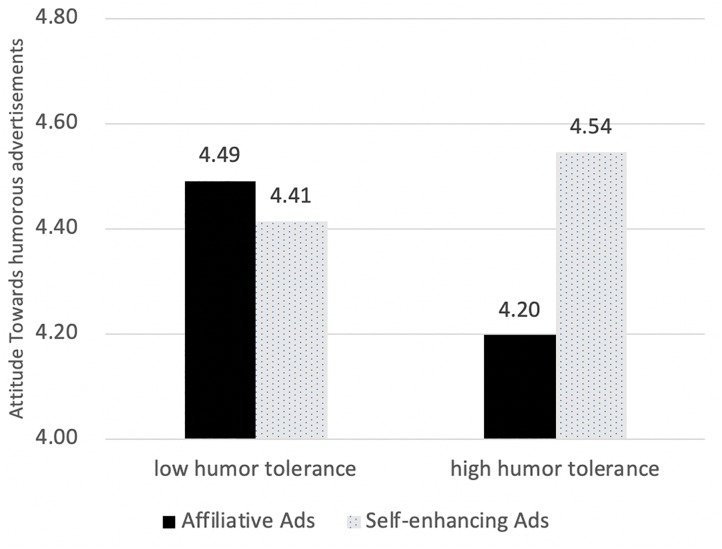
Attitude difference in advertising tactics with different levels of humor tolerance.

We also found a significant interaction of humor tolerance and brand nationality, *F*(1,382) = 3.915, *p* < 0.5 (Figure 4). Simple main effect analyses showed that audiences high in humor tolerance held more positive attitudes toward self-enhancing ads than affiliative ads [*F*(1,382) = 9.801, *p* < 0.01]; whereas Chinese audiences did not differ, *F*(1,382) = 0.363, *p* > 0.05.

### Uncertainty Avoidance as a Cultural Moderator

Uncertainty avoidance is another important cultural factor that has been shown to influence the way an audience perceive and react to humorous advertisements. We categorized participants into high and low uncertainty avoidant groups based on a mean level of uncertainty avoidance, and conducted a univariate analysis of covariance to test the prediction that audiences with different uncertainty avoidance hold different attitudes toward humorous advertisements depending on humor tactics and brand nationality, controlling for age, gender, and income. Table 3 displays the results in detail.

**Table 3:** Attitude model of cultural values above audience nationality.

Dependent variable: Attitude toward humorous advertisements															

	Model 1			Model 2			Model 3			Model 4			Model 5		
	B	SE	t	B	SE	t	B	SE	t	B	SE	t	B	SE	t
(Constant)	4.298	0.18	23.831	4.495	0.267	16.809	4.325	0.518	8.349	4.376	0.529	8.264	4.4	0.567	7.767
gender	0.005	0.097	0.05	0.015	0.097	0.149	0.054	0.095	0.567	0.063	0.096	0.651	0.056	0.097	0.581
age	−0.05	0.04	−1.234	−0.032	0.043	−0.742	−0.042	0.042	−1.013	−0.044	0.043	−1.023	−0.038	0.043	−0.883
incoming	0.143^∗^	0.061	2.333	0.144^∗^	0.061	2.347	0.168^∗∗^	0.06	2.798	0.17^∗∗^	0.06	2.819	0.174^∗∗^	0.061	2.845
Brand nationality				−0.232^∗^	0.095	−2.44	0.133	0.302	0.439	0.118	0.304	0.387	0.173	0.321	0.539
Advertising tactics				0.087	0.095	0.918	−0.074	0.305	−0.244	−0.097	0.308	−0.315	−0.16	0.317	−0.504
Audience nationality				−0.117	0.103	−1.131	−0.185	0.403	−0.46	−0.151	0.408	−0.369	−0.102	0.476	−0.214
Brand nationality^∗^ Advertising tactics							−0.074	0.185	−0.399	−0.061	0.187	−0.326	−0.077	0.189	−0.408
Brand nationality^∗^ Audience nationality							−**0.646^∗∗^**	**0.19**	−**3.404**	−**0.644^∗∗^**	**0.19**	−**3.387**	−**0.565^∗^**	**0.229**	−**2.464**
Advertising tactics^∗^ Audience nationality							**0.697^∗∗∗^**	**0.188**	**3.706**	**0.7^∗∗∗^**	**0.189**	**3.704**	**0.587^∗^**	**0.229**	**2.563**
Uncertainty avoidance										−0.033	0.099	−0.332	−0.204	0.414	−0.494
Humor tolerance										−0.061	0.111	−0.553	−0.085	0.463	−0.184
Humor tolerance^∗^ Brand nationality													−**0.083**	**0.221**	−**0.374**
Humor tolerance^∗^ Advertising tactics													**0.099**	**0.223**	**0.445**
Uncertainty avoidance^∗^ Advertising tactics													**0.201**	**0.198**	**1.015**
Uncertainty avoidance^∗^ Brand nationality													−**0.086**	**0.196**	−**0.439**
*F*	**2.086**			**2.363^∗^**			**4.332^∗∗∗^**			**3.568^∗∗∗^**			**2.693^∗∗^**		
Adj. *R*^2^	**0.008**			**0.020^∗^**			**0.071**			**0.067^∗∗∗^**			**0.061^∗∗^**		

*^∗^p < 0.05, ^∗∗^p < 0.01, and ^∗∗∗^p < 0.001.*

Result showed that the main effect of brand nationality was significant, *F*(1,382) = 7.297, *p* < 0.01. Audiences held a more positive attitude toward Chinese brands than United States brands, confirming Hypothesis 1.

**FIGURE 5:**
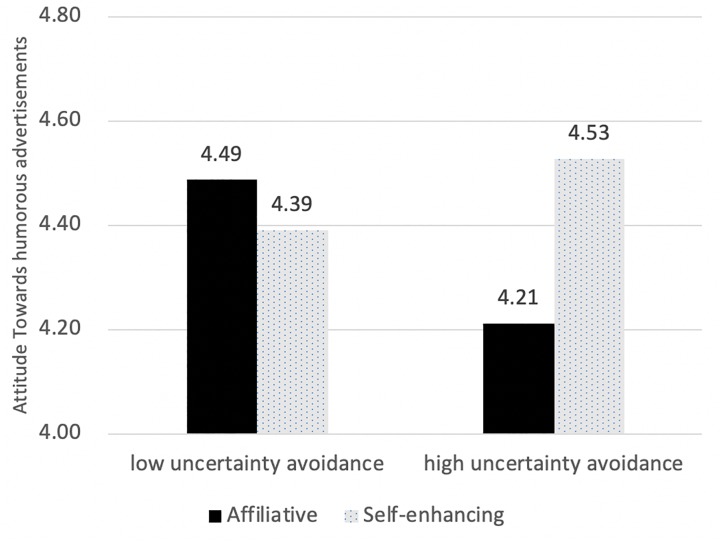
Attitude difference in advertising tactics with different levels of uncertainty avoidance.

As Hypothesis 5 suggested, uncertainty avoidance significantly interacted with humor tactics, *F*(1,382) = 4.588, *p* < 0.05 (Figure 5). Simple main effect analyses showed that a high uncertainty avoidant audience held more positive attitudes toward self-enhancing ads than affiliative ads [*F*(1,382) = 5.244, *p* < 0.05]; whereas a low uncertainty avoidant audience did not differentiate, *F*(1,382) = 0.514, *p* > 0.05.

**FIGURE 6:**
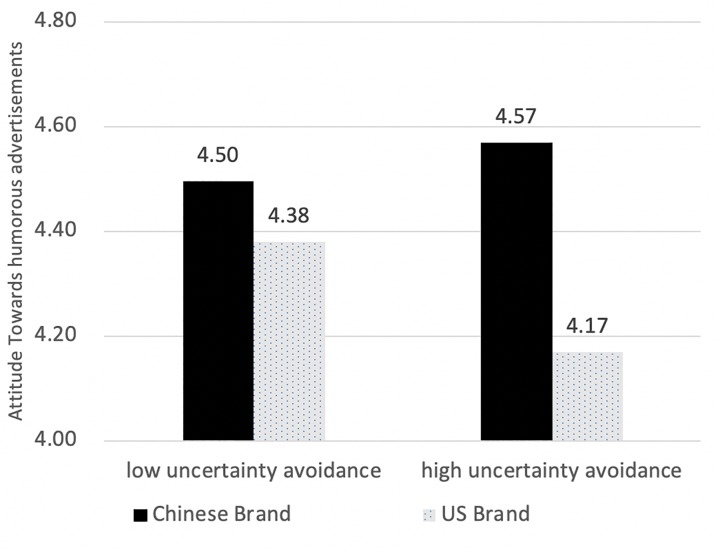
Attitude difference in brand nationality with different levels of uncertainty avoidance.

The interaction of uncertainty avoidance and brand nationality was marginally significant, *F*(1,382) = 2.208, *p* = 0.138 (Figure 6). Simple main effect analyses showed that a high humor tolerant audience held more positive attitudes toward Chinese brands than United States brands, *F*(1,382) = 8.471, *p* < 0.01; whereas low humor tolerant audiences did not differ, *F*(1,382) = 0.766, *p* > 0.05.

### Incremental Contribution of Cultural Values Above Audience Nationality

To test whether individual level variance in cultural values could fully explain and substitute the effect of ethnic cultural groups, we conducted a multilevel linear regression on attitudes toward humorous advertisement. Table 3 shows the results in detail. A model tested earlier in the MANCOVA explains 7.1% of the total variance in attitudes toward humor advertisement (Model 3). Taking humor tolerance and uncertainty avoidance into account don’t make any significant contribution, *F*(2,381) = 0.209, Δ*R*^2^ = 0.001, *p* > 0.05 (model 4). The same was true after interaction terms of cultural values and humor tactics, and cultural values and brand nationality were entered into the model, *F*(4,377) = 0.355, Δ*R*^2^ = 0.003, *p* > 0.05 (model 5). These results suggest that audience nationality related effects found in 4.1 could not be explained or substituted by individual variance in humor tolerance and uncertainty avoidance.

## General Discussion

Humor is an important appeal used in commercial advertising. In the era of globalization, how to carry out humorous advertisements in countries with different cultural backgrounds is a major challenge for enterprises. Answering this call, the current study explored the impact of characteristics of humorous advertisements on audience attitudes toward humorous advertisements in a cross-cultural research framework. The results suggest that the attitudinal differences between Chinese audiences and United States audiences do not lie at the aggregate level. In other words, we didn’t find a main effect of audience nationality. Instead, the differences lie in an audience’s sensitivity/responsiveness to characteristics of the ads. For example, United States audiences showed a strong preference for ads of Chinese brand to those of United States brands, whereas, Chinese audiences did not differentiate them. Whereas United States audiences preferred ads using self-enhancing tactics to those using affiliative tactics, again Chinese audiences didn’t differentiate. These findings coincide with the “culture-bound” humor use effect as suggested by Kuiper et al. (2010), such that a preference for self-enhancing humor tactics is more prevalent in the North American individualistic culture.

Given past research which suggests that, “…between country differences are not so large as to preclude successful use of humor in standardized advertising” (Gregory and Crawford, 2011, p. 239), we also explored whether individual differences in cultural values could account for the effect of audience nationality. Similar interaction patterns were obtained when we replaced audience nationality with humor tolerance, and uncertainty avoidance, respectively. Specifically, the results of a high humor tolerant audience and a high uncertainty avoidant audience parallel that of United States audiences, such that they showed significant preferences for ads of Chinese brands and with self-enhancing tactics. Whereas the result of low humor tolerant audiences and low uncertainty avoidant audiences parallel that of Chinese audience, such that they showed no preference for either brand nationalities or for either humor tactics. We further conducted a multilevel linear regression to test incremental contributions of cultural values on and above audience nationality related effects. The results showed that taking humor tolerance and uncertainty avoidance into account does not make any significant contribution. The same is true after interaction terms of cultural values and humor tactics, and cultural values and brand nationality were entered into the model. More importantly, audience nationality related effects remained significant, suggesting that differences embedded in culture groups, as indicated by audience nationality, could not be explained or substituted by individual variance in humor tolerance and uncertainty avoidance.

The current research offers important managerial implications by highlighting the attitudinal differences between Chinese and Americans. That is, the differences do not lie at the aggregate level; instead, whereas the Chinese are not responsive toward advertisements with different brand nationalities and humor tactics, Americans are rather sensitive. This sensitivity is cultivated by intensive and frequent exposure to modern advertising commercials. In fact, as early as 29 years ago, “on any given day, the average American is exposed to about 300 ad messages. That is 9,900 a month, or 109,500 a year” (McCarthy, 1991). Therefore, we suggest that for humor appeals and other characteristics of an ad to take effect in countries that show lower sensitivity or responsiveness, cultivating a modern commercial culture takes priority over the field. This is especially true when MNCs are expanding to overseas markets.

This study is not without limitations. First, we built our research framework on the assumption that Chinese and American people share a common understanding of a humor, without directly testing it. In fact, this is true only for the types of humor that employ punchlines that are relatively universal (Robert, 2005). Given that knowledge about products and brand nationalities shared by one country may differ tremendously from that of another country, it is highly probable that differences in attitudes are caused by differences in understanding. Second, although we used a 2 × 2 × 2 semi-experimental design, we did not find a significant three-way interaction or a two-way interaction of brand nationality and humor tactics, questioning the value of the brand nationality × humor tactics orthogonal design. Last but not least, although we developed our hypotheses based heavily on the distinction between collective and individualistic self-construal, we did not directly test them. As a potential mediator, collective and individualistic self-construal may covary with cultural values, such as humor tolerance and uncertainty avoidance, to some extent, but may not perfectly overlap. This might be the reason that we failed to find any mediation roles played by humor tolerance or by uncertainty avoidance.

Economic globalization has provided cross-cultural research on humorous advertising broader opportunities than ever, and future research could focus on the following aspects. Firstly, millennial young adults are more exposed and sensitive to social media, meaning it is highly probable that their cultural values are shaped differently from their parents, and the sub-culture they share could influence their reactions and attitudes toward humorous advertisements. Future research could compare participants from different generations and observe the effect of differences between sub-cultures on attitudes toward humorous advertisements. Second, future research could investigate other variables that might affect audience attitudes toward advertising, such as social media influences and esthetic stimuli (Buijzen and Valkenburg, 2004). Furthermore, in recent years, online advertising has become more widespread around the globe, and future research can be extended to online display advertisements and online video advertisements.

## Ethics Statement

Our study was approved by the Ethics Review Board of Business School of Central University of Finance and Economics. All participants signed an informed consent form.

## Author Contributions

All authors conceptualized the manuscript. YW and JL wrote the first complete draft. SL contributed additional writing and further analysis, JT and JZ contributed data collection and analysis, and all authors edited the manuscript and approved the final version.

## Conflict of Interest Statement

The authors declare that the research was conducted in the absence of any commercial or financial relationships that could be construed as a potential conflict of interest.
